# Effect of fasting and subsequent refeeding on the transcriptional profiles of brain in juvenile *Spinibarbus hollandi*

**DOI:** 10.1371/journal.pone.0214589

**Published:** 2019-03-28

**Authors:** Yang Yang, Huiqiang Zhou, Hu Shu, Dongming Zhong, Mingqing Zhang, Jun Hong Xia

**Affiliations:** 1 School of Life Science, Guangzhou University, Guangzhou, China; 2 State Key Laboratory of Biocontrol, Institute of Aquatic Economic Animals and Guangdong Provincial Key Laboratory for Aquatic Economic Animals, College of Life Sciences, Sun Yat-Sen University, Guangzhou, PR China; National Cheng Kung University, TAIWAN

## Abstract

Starvation is a common stress in fish. The underlying molecular mechanisms associated with growth depression caused by feeding restriction and compensatory growth are not well understood. We investigated the effect of fasting and refeeding on the transcriptome profiles of brain in juvenile *S*. *hollandi* using RNA-seq. A total of 4.73 × 10^8^ raw reads were obtained from nine brain samples. *De novo* transcriptome assembly identified 387,085 unigenes with 2.1×10^9^ nucleotides. A total of 936 annotated unigenes showed significantly differential expression among the control, fasting, and fasting-refeeding groups. The down-regulated differentially expressed genes (DEGs) during fasting were mainly associated with cell cycle, DNA replication, and mitosis. The up-regulated DEGs were mainly related to glucose and lipid metabolism, material transportation, and transcription factors. Most decreased DEGs during fasting were restored to normal levels after refeeding. Comparing with the control group, genes associated with protein synthesis, stimulus response, and carbohydrate metabolism were significantly over-expressed and pro-opio melanocortin (POMC) was down-regulated during the refeeding period. In conclusion, fish mobilized stored energetic materials and reduced energy consumption to prolong survival during fasting. After refeeding, the down-regulation of DEGs, e.g., POMC may be associated with compensatory growth. Up-regulation of DEGs related to ribosomal protein, stimulus response, and carbohydrate metabolism may contribute to eliminate negative effect of starvation on brain. This study provided the first transcriptome data related with impact of short-time starvation and refeeding in *S*. *hollandi* brains.

## Introduction

Starvation is a common stress in fish. Extreme aquatic conditions, such as unsuitable temperature, hypoxia, and high population density, can affect the feeding behavior of aquatic animals. The starvation condition is strongly associated with the mobilization of the body’s energy reserves, which are mainly carbohydrates, fats, and proteins[1]. During starvation, organisms usually maintain metabolic homeostasis by changing their enzymatic activities and hormone levels, and activating various physiological and biochemical adaptive mechanisms[2]. In previous studies, the impacts of starvation on transcriptome profiles have been investigated in several fish species, such as the yellow croaker (*Larimichthys crocea*)[3], rainbow trout (*Oncorhynchus mykiss*)[4], and channel catfish (*Ictalurus punctatus*)[5]; however, these reports mainly focused on liver and muscle tissues[3,4].

Compensatory growth is defined as the physiological process of accelerated growth when food intake is restored following a period of food deprivation[6]. The phenomenon is ubiquitous in teleosts such as European minnow (*Phoxinus phoxinus*) [7], gibel carp (*Carassius auratus gibelio*)[8], barramundi (*Lates calcarifer*)[9], channel catfish (*Ictalurus punctatus*)[10], tongue sole (*Cynoglossus semilaevis*)[11], and Atlantic salmon (*Salmo salar*)[12]. Information regarding the biological and molecular mechanisms of modulating the exaggerated growth phenotype remains limited. Recently, studies on fine flounder (*Paralichthys adspersus*)[13], grass carp (*Ctenopharyngodon idella*)[14], and rainbow trout[4], have revealed changes in the transcriptome profile of muscle during the fasting and refeeding period. He et al. described the global gene expression patterns of liver following compensatory growth in grass carp and identified several genes potentially associated with compensatory growth [14].

*Spinibarbus hollandi*, an endemic Cyprinidae species in southeastern China, is mainly distributed in the provinces of Anhui, Hubei, Hunan, Fujian, Guangdong, and Guangxi. *S*. *hollandi* is an easy-to-raise omnivore, and has already attracted increasing attention owing to its high nutritional and medicinal value. However, the low growth rate has severely limited its farming[15]. In recent years, researchers attempted to enhance the growth rate of *S*. *hollandi* by improving the rearing condition and via cross-breeding. We have developed molecular markers associated with growth traits in *S*. *hollandi*[15] and recently observed the compensatory growth process in this species. Currently, studies on the effects of fasting on the growth rate and the compensatory growth of *S*. *hollandi* are rare. The underlying molecular mechanisms associated with growth depression caused by feeding restriction and compensatory growth are not well understood.

The next-generation sequencing technology has revolutionized the field of genomics. RNA-Seq can be used to analyze the transcriptome of non-model species without requiring their genomic information [16–20]. Brain plays an important role in maintaining energy balance during the fasting and refeeding period. The aim of this study was to investigate the changes of transcriptome profile of *S*. *hollandi* brain in response to fasting and refeeding. Our study provides an important dataset for understanding the molecular mechanism of starvation response and compensatory growth in fish.

## Materials and methods

### Fasting treatment and sampling

*S*. *hollandi* fish at about one-month-old were obtained from Shaoguan Fisheries Research Institute, Guangdong Province, China. The fish were sexually immature and had an approximate weight of 3–5 cm. 210 fish were equally distributed to seven aquariums containing 100 L of continuous flow, filtered water with aeration. The fish were housed at 26°C with a 14:10 h light/dark photoperiod. The fish were fed with a commercial pellet containing 35% crude protein, 4% crude fat, 14% crude fiber, 14% crude ashes, and 12% humidity (Qicai Pet Products Co., Ltd., Guangzhou, China), twice daily to apparent satiation at 7:00 am and 5:00 pm, for a 2-week acclimation. Of the seven aquariums, four were served as control groups, one was used as the fasting group and the remaining two aquariums were used as the refeeding groups.

Fish in the control groups were fed to apparent satiation everyday as mentioned above. The fish were sequentially sampled on day 0 (C0), day 7 (C7), day 14 (C14), and day 40 (C40). Fish in the fasting group were deprived of feed for 7 days, and then sampled (F7). The fish in the refeeding groups were fed twice per day to satiation after 7 days of fasting. The fish were sampled after 7 (R7) and 33 days refeeding (R33). All seven groups were then anesthetized by an overdose (100 ppm) of eugenol (Sangon Biotech (Shanghai) Co., Ltd., Shanghai, China). The body weights of each group were measured prior to sampling (n = 8). Brain tissues were rapidly isolated, immediately frozen in liquid nitrogen, and stored at -80°C.

### RNA isolation

Total RNA of brain tissues was extracted using RNAiso reagents (Takara Biomedical Technology Co., Ltd., Dalian, China), following the manufacturer’s instructions. The quantity and quality of RNA samples were determined using Epoch Microplate Spectrophotometer (BioTek Instruments, Inc., USA), Agilent 2100 instruments and electrophoresis using 1% agarose gel. RNA samples with 28S/18S ratio >1.0, optical density (OD) 260/280 values >1.8 and <2.0, and RNA yield > 5 μg were used for subsequent transcriptome analysis.

### cDNA library preparation and sequencing

RNA samples of the C7, F7, and R7 groups were used for RNA-seq analysis. To minimize the variation among individuals, equal quantities of RNA from 5 samples belonging to the same group were pooled, and 3 replicates of mixed RNA pools were prepared for each group. A total of 9 RNA pools were used for cDNA library preparation. mRNA was purified from total RNA using oligo (dT) magnetic beads and fragmented using fragmentation buffer. First-strand cDNA was synthesized using SuperScript II Reverse Transcriptase (Applied Biosystems Ltd., USA) followed by second strand generation using the components dNTPs, buffer, DNA polymerase I, and RNase H. After purification, adapters were ligated to the cDNA, and then enriched using PCR. The nine cDNA libraries were sequenced on an Illumina-Hiseq 2000 platform using the 150 bp paired-end approach.

### Data processing and analysis

Clean reads were generated by removing low-quality reads reads containing adapter and ploy-N from raw data. Clean reads were *de novo* assembled using Trinity software version 2.5.1 [21]. The assembled transcripts were identified using Blast+ (version: ncbi-blast-2.2.28+) against NCBI non-redundant (Nr, e-value <1e^-5^), Clusters of Orthologous Groups of proteins (COG, e-value <1e^-3^), Kyoto Encyclopedia of Genes and Genomes (KEGG, e-value <1e^-5^), and Swiss-Prot databases (e-value <1e^-5^). Gene ontology (GO) annotation was performed using Blast2GO with an e-value threshold of 1e^-6^.

Total number of mapped reads for each transcript was determined and normalized, to calculate the expected number of Fragment Per Kilobase of transcript sequence per million base pairs sequenced (FPKM) by using RSEM V1.2.15 [22]. Differential expression analyses of three groups (C7, F7, and R7) were performed using DESeq R [23]. The adjusted *p*-value (*p-adj*) with a cut-off of 0.05 was used to identify significant differentially expressed genes (DEGs). Pathway enrichments of DEGs were analyzed using the KEGG database.

### RNA-seq validation by quantitative real-time PCR analysis

Six DEGs (CDC6, CDC20, MCM2, DHCR7, FABP7, HSP70) belonging to different functional classification were selected and analyzed using the software FasParser [24] to identify gene expression changes in seven groups (C0, C7, C14, C40, F7, R7, and R33) through quantitative real-time PCR (qRT-PCR). A total of 10 DEGs including the above six genes, and TFRC1, TUBB5, MCH1, and ACSBG2, were used to verify RNA-seq results. It was performed by detecting expression changes of these DEGs during fasting (C7 vs. F7) and refeeding (C7 vs. R7) using qRT-PCR. Three biological replicates were used for each RNA sample and the beta-actin gene was used as an internal control. Primers used for qRT-PCR analysis are shown in S1 Table. The qRT-PCR analysis was performed using ABI 7000 platform with 20 μL reactions containing the following components: 100 ng of cDNA, 10 μL Power SYBR Green PCR Master Mix (Vazyme Biotech, Nanjing, China), 0.3 μL of each primer (10 μmol/L), and 7.4 μL double-distilled water. The reaction procedure followed by 95°C for 30 s, 40 cycles at 95°C for 10s and 60°C for 30s. After amplification, a melting curve was obtained by default processes in ABI7000. All samples were analyzed in triplicate, and fold changes were calculated using the comparative Ct method (2^−ΔΔCt^ method) [25]. All data are provided in terms of relative mRNA expression as mean ± S.E. (n = 3).

## Results

### Changes of body weights during fasting and refeeding periods

**Fig 1:**
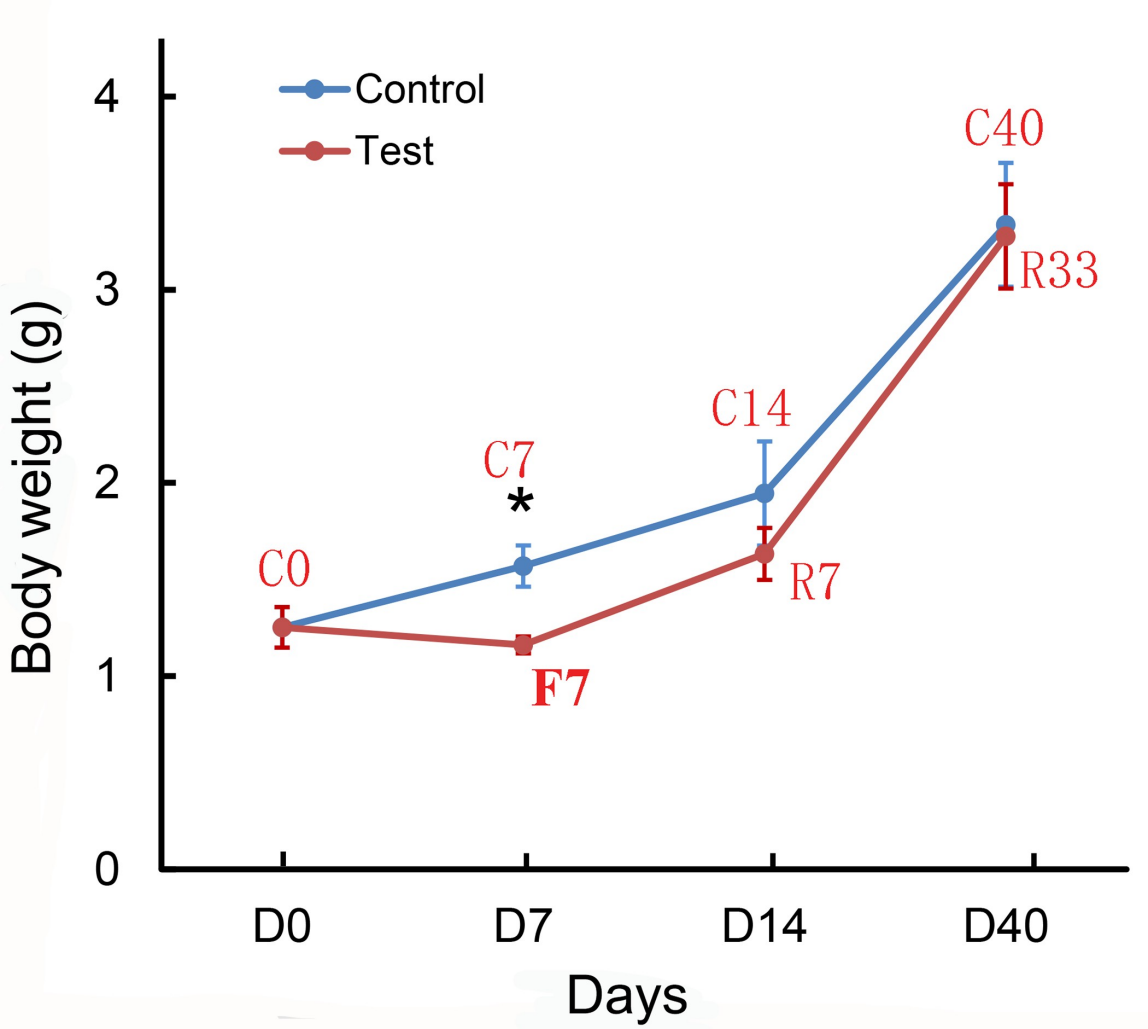
Growth curves of *Spinibarbus hollandi* under continuous feeding and fasting-refeeding. Asterisk indicated significant difference between two groups (*p* < 0.05); blue line represented the growth curve for the continuous feeding groups; red line indicated the growth curve for the fasting-refeeding groups.

The body weights of fish in the control groups (C7, C14, and C40) and the test groups (F7, R7, and R33) were measured. The growth curves for these groups were shown in Fig 1. At the end of 7-day fasting period, the body weight of C7 and F7 were 1.57 g and 1.16 g, respectively. There was a significantly difference in body weights between groups (*p* < 0.05). After 7-day refeeding, the body weight of C14 and R7 were 1.94 g and 1.63 g. After 40 days, the body weights for C40 and R33 was 3.34g and 3.28 g, respectively. However, no significant difference between groups was detected at the same time points.

### RNA-seq data analysis

Through high-throughput paired-end sequencing, 4.93 × 10^8^ raw reads (150 bp) were yielded from nine brain samples. After removing low-quality sequences, 4.73 × 10^8^ clean reads with 71.01 Gb were generated. The Q20 and Q30 of all samples were above 96.27% and 90.97%, respectively. The sequence data were deposited in the NCBI SRA database, under the accession number, SRP156354.

After *de novo* assembly, 864,488 transcripts (ranging from 201 to 23,693 bp) were generated. The longest transcript of a gene was defined as a unigene for further analysis. A total of 387,085 unigenes with 2.1×10^9^ nucleotides were obtained, with an average length of 568 bp. The N50 and N90 were 752 and 254 bp, respectively.

A total of 349,336 (90.24%) unigenes could be annotated in at least one of the following database: Nr, Nt, Pfam, KOG, Swiss-Prot, KEGG, and GO. A total of 10,438 (6.65%) unigenes were annotated in all these databases. Among of all unigenes, 76,988 (19.88%) unigenes were matched in the Nr database. Of which, nearly 58.6% could be annotated with sequences from *Danio rerio*, followed by those from *Astyanax mexicanus* (5.5%), *Clupea harengus* (2.8%), *Mus musculus* (2.4%), and *Oncorhynchus mykiss* (2.3%).

Functional prediction and classification of all unigenes were performed using the GO database. A total of 71,164 unigenes were classified into 56 GO terms, including 10 molecular function terms, 20 cellular component terms, and 26 biological process terms. Cellular process (39,913), binding (37,484), single-organism process (32,048), metabolic process (31,836), and cell (24,674), were the five most abundant functional terms at the second GO level (S1 Fig).

Furthermore, all unigenes were aligned to COG database for phylogenetic classification. In total, 22,886 unigenes were divided into 26 functional categories (S2 Fig). The largest category was signal transduction mechanisms with 4,653 unigenes followed by general function prediction (3,511), posttranslational modification, protein turnover, chaperones (2,283), intracellular trafficking, secretion, and vesicular transport (1,725).

To interpret the biological pathways, we annotated all assembled unigenes in the KEGG database, and 39,265 unigenes were matched in level 2 of the KEGG pathway. Signal transduction (6744), endocrine system (3302), cellular community (2976), immune system (2643), and nervous system (2432) were the five most significant matched catalogs in the KEGG pathway (S2 Table).

### Identification of DEGs between control and fasting group

A total of 936 annotated unigenes showed significantly differential expression among the C7, F7, and R7 groups. Of the DEGs, 464 were significantly expressed between C7 and F7 (fasting period), 491 were significantly expressed between F7 and R7 (refeeding period), and 230 were significantly expressed between C7 and R7.

After fasting, of the 464 DEGs, 169 and 295 genes were up-regulated and down-regulated, respectively. Most of the genes associated with cell division were significantly down-regulated (Table 1). These genes were related to various key proteins involved in the cell cycle (*CCNA2*, *CDC20*, *CDC25A*, *CDC6*, *CDK1*, *CDK2*, *CHEK2*, *E2F3*, *PLK1*, and *PTTG1*), DNA replication (*MCM2*, *MCM3*, *MCM4*, *MCM5-B*, *MCM7-A*, *PCNA*) and mitosis (*ESPL1*, *MAD2L1*, *NCAPD2*, *NCAPG*, *MAD2L1*, *SMC2*, *and SMC4*).

**Table 1:** Down-regulated DEGs involved in cell division after 7-day fasting.

Gene name	Gene ID	Gene description	log_2_FC	*padj*
CCNA2	TRINITY_DN98393_c2_g1	Cyclin-A2	1.235	1.7884E-02
CDC20	TRINITY_DN101219_c0_g1	Cell division cycle protein 20 homolog	1.2824	2.6243E-06
CDC25A	TRINITY_DN97504_c0_g1	M-phase inducer phosphatase 1	1.3734	1.1450E-05
CDC6	TRINITY_DN100724_c0_g3	Cell division control protein 6 homolog	1.3301	1.4772E-02
CDK1	TRINITY_DN90363_c1_g2	Cyclin-dependent kinase 1	1.2218	7.9794E-05
CDK2	TRINITY_DN73824_c1_g1	Cyclin-dependent kinase 2	1.1128	1.4598E-02
CHEK2	TRINITY_DN99705_c1_g2	Serine/threonine-protein kinase Chk2	1.4801	2.7848E-02
E2F3	TRINITY_DN109456_c1_g1	Transcription factor E2F3	1.3032	3.6578E-03
ESPL1	TRINITY_DN110892_c9_g2	Separin	1.5113	1.0703E-02
MAD2L1	TRINITY_DN84178_c0_g1	Mitotic spindle assembly checkpoint protein MAD2A	1.0504	4.9257E-02
MCM2	TRINITY_DN107178_c1_g1	DNA replication licensing factor mcm2	1.6036	5.2371E-15
MCM2	TRINITY_DN107178_c1_g4	DNA replication licensing factor mcm2	1.7659	7.7048E-03
MCM3	TRINITY_DN86555_c1_g1	DNA replication licensing factor MCM3	1.3235	6.8196E-08
MCM4	TRINITY_DN108416_c1_g1	DNA replication licensing factor mcm4	1.4637	5.6243E-09
MCM4	TRINITY_DN108416_c1_g2	DNA replication licensing factor mcm4	1.5125	3.2801E-06
MCM5-B	TRINITY_DN108607_c2_g1	DNA replication licensing factor mcm5-B	1.6463	6.7690E-08
MCM7-A	TRINITY_DN94788_c0_g1	DNA replication licensing factor mcm7-A	0.86554	2.6866E-05
NCAPD2	TRINITY_DN109869_c3_g2	Condensin complex subunit 1	0.9826	1.5797E-03
NCAPG	TRINITY_DN107734_c1_g2	Condensin complex subunit 3	1.6892	5.3557E-09
NCAPG	TRINITY_DN107734_c1_g3	Condensin complex subunit 3	1.368	9.6760E-03
PCNA	TRINITY_DN97153_c1_g2	Proliferating cell nuclear antigen	0.85759	4.0344E-05
PLK1	TRINITY_DN101420_c0_g1	Serine/threonine-protein kinase PLK1	1.5956	1.9129E-03
PLK1	TRINITY_DN101420_c0_g3	Serine/threonine-protein kinase PLK1	1.3167	5.6127E-05
PTTG1	TRINITY_DN85037_c0_g1	Securin	1.1932	1.7091E-02
SMC2	TRINITY_DN107599_c0_g1	Structural maintenance of chromosomes protein 2	1.4581	5.9764E-11
SMC4	TRINITY_DN108206_c1_g1	Structural maintenance of chromosomes protein 4	0.90865	8.2643E-05

**Fig 2:**
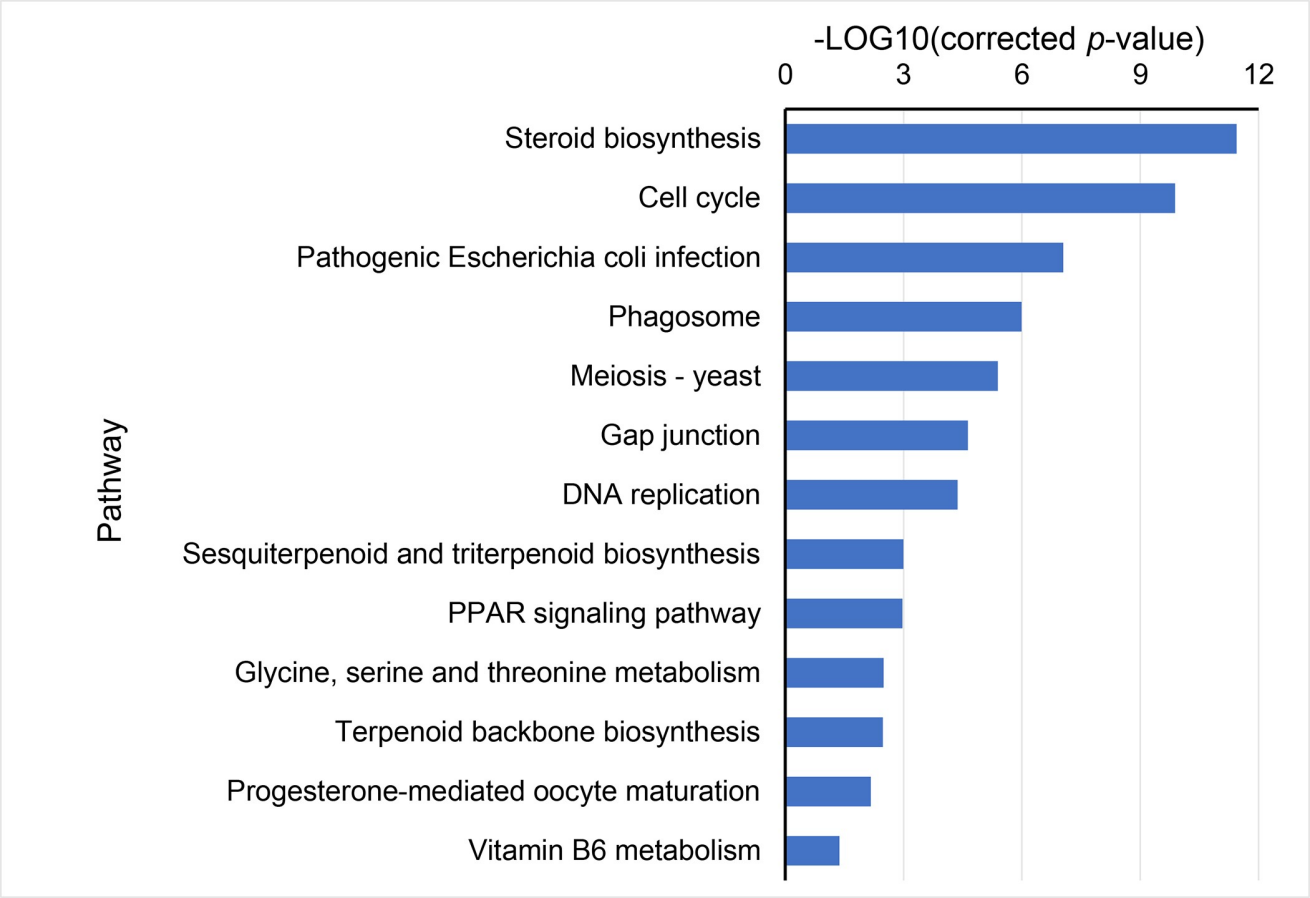
Classification of down-regulated DEGs during fasting according to KEGG database.

Except the above catalogs, KEGG pathway analysis identified that the down-regulated genes were also enriched in other pathways, such as steroid biosynthesis, pathogenic *Escherichia coli* infection, phagosome, gap junction, and sesquiterpenoid and triterpenoid backbone biosynthesis (Fig 2).

The up-regulated DEGs were annotated and analyzed (S3 Table). We observed several up-regulated DEGs involved in regulating glucose and lipid metabolism (*PDK4*, *ADIPOR1*, *ARRDC3*, *PMM1*, *ZBTB16*, and *BBOX 1*), material transportation (*SLC43A2*, *SLC16A3*, *SLC19A3*), atrophy (*FBXO32*), protein synthesis (*EEF2K*, *PRKCA*), and autophagy (*GABARAPL1*, *TP53INP2*) (Table 2).

**Table 2:** Up-regulated DEGs after 7-day fasting in brain.

Gene name	Gene ID	Gene description	log2FC
PDK4	TRINITY_DN95752_c2_g2	pyruvate dehydrogenase kinase isoenzyme 4	1.8265
ADIPOR1	TRINITY_DN92796_c0_g1	Adiponectin receptor protein 1	0.7845
ARRDC3	TRINITY_DN107064_c1_g2	Arrestin domain-containing protein 3	0.84685
PMM1	TRINITY_DN101868_c1_g2	Phosphomannomutase 1	1.0098
ZBTB16	TRINITY_DN106696_c2_g1	Zinc finger and BTB domain-containing protein 16	0.76865
SLC16A3	TRINITY_DN107827_c3_g1	Monocarboxylate transporter 4	0.79206
SLC19A3	TRINITY_DN89387_c3_g3	Thiamine transporter 2	0.94927
PDE5A	TRINITY_DN95368_c1_g1	cGMP-specific 3',5'-cyclic phosphodiesterase	0.90502
FBXO32	TRINITY_DN93305_c1_g1	F-box only protein 32	1.2457
EEF2K	TRINITY_DN106107_c1_g1	Eukaryotic elongation factor 2 kinase	1.1637
PRKCA	TRINITY_DN107660_c5_g2	Protein kinase C alpha type	0.57357
GABARAPL1	TRINITY_DN75369_c2_g1	Gamma-aminobutyric acid receptor-associated protein-like 1	0.86148

### Identification of DEGs between fasting and refeeding group

**Fig 3:**
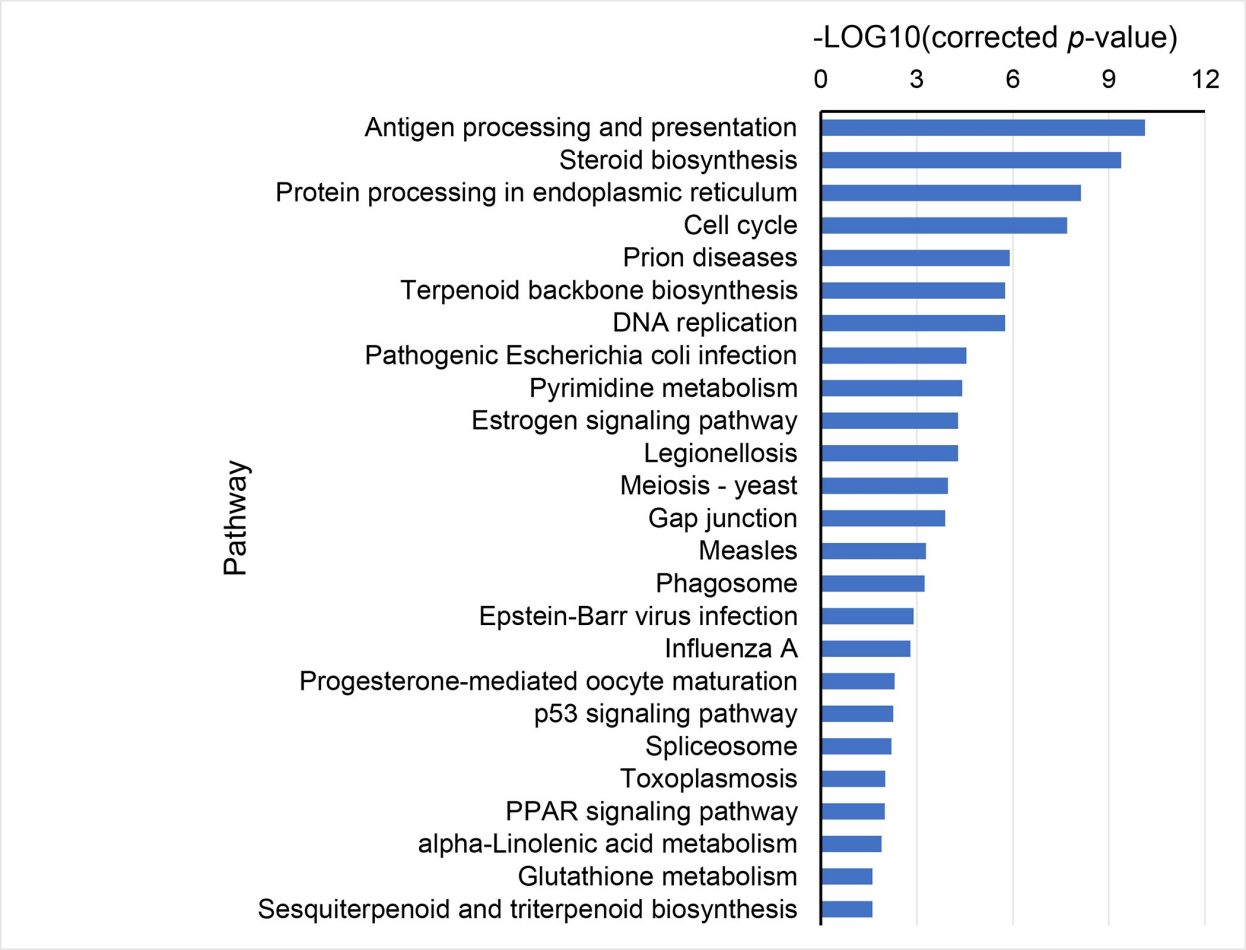
Classification of up-regulated DEGs after 7-day refeeding according to KEGG database.

After 7-day refeeding, of the 491 DEGs, 374 and 117 genes were up-regulated and down-regulated, respectively. The up-regulated DEGs were remarkably enriched in antigen processing and presentation, cell cycle, steroid biosynthesis, protein processing in endoplasmic reticulum, DNA replication, mitosis, pathogenic *E*. *coli* infection, and terpenoid backbone biosynthesis (Fig 3).

The down-regulated DEGs were identified and annotated (S4 Table). These genes included some of the genes up-regulated during the fasting period, such as *FBXO32*, *EEF2K*, *PDK4*, *ZNF778*, *ZBTB16*, *SLC43A2*, *SLC19A3*, *SLC16A3*, *PMM1*, and *GABARAPL1*. The ATP-binding cassette transporters (*ABCA1*, *ABCC5*), *IGFBP1*, *IRS2*, *JARID2*, *ACACB*, *CHKA*, *PDK2*, *PPARA* were also down-regulated.

### Identification of DEGs between control and refeeding group

DEGs between the control and refeeding group were identified and annotated (S5 Table). DEGs encoding ribosomal protein (*RPS11*, *RPS12*, *RPS13*, *RPS15*, *RPLP0*, *RPLP2*, *RPL10A*, *RPL12*, *RPL13*, and *RPL13A*), the heat shock protein (HSP) family (*HSP70*, *HSC71*, *HSC71*, *HSPA8*, *HSP90A1*, and *HSP90AA1*) and enzymes associated with glycolysis and gluconeogenesis (*LDHB*, *TPI1*, *GAPDH*, *PGAM1*, *LDHA*, *PGK1*, *ALDOA*, *GPI*) were all up-regulated (Table 3). The expression of *MT-ND5*, *MT-CO1*, *ITM2A*, *ACTG1*, and *IL32* were also significantly increased. Several DEGs associated with the electron transport chain (*MT-CO3*, *MT-ND3*, *MT-ND6*) were down-regulated, and the expression of *GTF2IRD2*, *JARID2*, *JARID2B*, *POMCA*, *STMN2*, and *UCHL1* were also significantly decreased.

**Table 3:** Up-regulated genes in the refeeding group comparing with the control group.

Gene name	Gene ID	Gene description	log2FC
Ribosomal protein			
RPSA	TRINITY_DN88004_c0_g3	40S ribosomal protein SA	4.535942951
RPS9	TRINITY_DN94296_c1_g2	40S ribosomal protein S9	4.18762245
RPS8	TRINITY_DN69062_c0_g1	40S ribosomal protein S8	2.33022993
RPS7	TRINITY_DN87535_c1_g2	40S ribosomal protein S7	5.265127258
RPS5	TRINITY_DN80306_c3_g4	40S ribosomal protein S5	3.929266576
RPS3A	TRINITY_DN77584_c1_g3	40S ribosomal protein S3a	4.032269037
RPS3	TRINITY_DN94897_c1_g6	40S ribosomal protein S3	4.308172588
RPS3	TRINITY_DN94897_c1_g5	40S ribosomal protein S3	3.255821893
RPS25	TRINITY_DN98335_c1_g1	40S ribosomal protein S25	4.757039305
RPS24	TRINITY_DN75798_c3_g1	40S ribosomal protein S24	2.163325301
RPS21	TRINITY_DN69959_c0_g2	40S ribosomal protein S21	3.569696459
RPS20	TRINITY_DN96702_c4_g9	40S ribosomal protein S20	3.858889849
RPS2	TRINITY_DN82419_c5_g1	40S ribosomal protein S2	6.7053945
RPS2	TRINITY_DN82419_c5_g2	40S ribosomal protein S2	3.589301509
RPS18	TRINITY_DN90356_c0_g5	40S ribosomal protein S18	5.417252941
RPS16	TRINITY_DN49145_c0_g2	40S ribosomal protein S16	4.748258981
RPS15A	TRINITY_DN102334_c0_g1	40S ribosomal protein S15a	3.784354695
RPS15	TRINITY_DN80664_c0_g4	40S ribosomal protein S15	4.829265745
RPS13	TRINITY_DN72902_c0_g2	40S ribosomal protein S13	3.677167149
RPS12	TRINITY_DN79746_c3_g1	40S ribosomal protein S12	4.387961359
RPS11	TRINITY_DN99507_c12_g4	40S ribosomal protein S11	4.506353942
RPLP2	TRINITY_DN102307_c1_g5	60S acidic ribosomal protein P2	3.712851556
RPLP0	TRINITY_DN81984_c0_g1	60S acidic ribosomal protein P0	1.534808896
RPL9	TRINITY_DN71792_c0_g2	60S ribosomal protein L9	3.867069267
RPL8	TRINITY_DN82367_c6_g1	60S ribosomal protein L8	6.943147623
RPL7	TRINITY_DN85697_c1_g1	60S ribosomal protein L7	4.733797505
RPL6	TRINITY_DN50400_c0_g1	60S ribosomal protein L6	4.861227838
RPL4	TRINITY_DN68389_c0_g1	60S ribosomal protein L4	5.294078581
RPL39	TRINITY_DN59795_c0_g1	60S ribosomal protein L39	4.060313367
RPL38	TRINITY_DN87593_c1_g5	60S ribosomal protein L38	3.262147471
RPL36	TRINITY_DN54848_c0_g2	60S ribosomal protein L36	3.633289825
RPL35	TRINITY_DN85158_c0_g1	60S ribosomal protein L35	4.190676949
RPL32-PS	TRINITY_DN89995_c3_g4	60S ribosomal protein L32	3.419806477
RPL31	TRINITY_DN65558_c0_g1	60S ribosomal protein L31	3.867236433
RPL3	TRINITY_DN93473_c1_g3	60S ribosomal protein L3	5.685872966
RPL28	TRINITY_DN8069_c0_g1	60S ribosomal protein L28	4.38506942
RPL27A	TRINITY_DN68249_c0_g1	60S ribosomal protein L27a	4.642412735
RPL26	TRINITY_DN92477_c0_g2	60S ribosomal protein L26	4.480991296
RPL18A	TRINITY_DN75517_c3_g5	60S ribosomal protein L18a	6.229154361
RPL18	TRINITY_DN65120_c0_g1	60S ribosomal protein L18	4.311837585
RPL17	TRINITY_DN64100_c0_g1	60S ribosomal protein L17	3.933855363
RPL15	TRINITY_DN76924_c1_g5	60S ribosomal protein L15	2.389800649
RPL13A	TRINITY_DN72454_c1_g2	60S ribosomal protein L13a	5.651412321
RPL13	TRINITY_DN97764_c3_g1	60S ribosomal protein L13	3.503720206
RPL12	TRINITY_DN83096_c3_g3	60S ribosomal protein L12	5.010585117
RPL10A	TRINITY_DN85223_c3_g1	60S ribosomal protein L10a	3.896006318
Heat shock protein family			
HSPA8	TRINITY_DN100126_c1_g2	Heat shock cognate 71 kDa protein	0.974477412
HSP90AA1	TRINITY_DN89892_c3_g3	Heat shock protein HSP 90-alpha	0.971776669
HSP90A1	TRINITY_DN109438_c3_g6	Heat shock protein HSP 90-alpha 1	0.842457828
HSP90A1	TRINITY_DN89892_c3_g1	Heat shock protein HSP 90-alpha 1	0.799479292
HSP70	TRINITY_DN111407_c8_g7	Heat shock 70 kDa protein	1.041022237
HSP70	TRINITY_DN111407_c8_g5	Heat shock 70 kDa protein 1	1.012967973
HSC71	TRINITY_DN84773_c1_g3	Heat shock cognate 70 kDa protein	0.816312679
HSC71	TRINITY_DN90379_c0_g3	Heat shock cognate 70 kDa protein	0.742926524
DNAJA4	TRINITY_DN98506_c3_g1	DnaJ homolog subfamily A member 4	1.352175205
glycolysis and gluconeogenesis			
ALDOA	TRINITY_DN93020_c1_g10	Fructose-bisphosphate aldolase A	3.825625389
GAPDH	TRINITY_DN73283_c0_g1	Glyceraldehyde-3-phosphate dehydrogenase	2.767387832
GPI	TRINITY_DN96702_c4_g7	Glucose-6-phosphate isomerase	4.875604882
LDHA	TRINITY_DN84691_c6_g1	L-lactate dehydrogenase A chain	7.697424007
LDHB	TRINITY_DN21161_c0_g1	L-lactate dehydrogenase B chain	3.711726558
PGAM1	TRINITY_DN79644_c1_g1	Phosphoglycerate mutase 1	6.856235293
PGK1	TRINITY_DN85707_c4_g2	Phosphoglycerate kinase 1	4.479693518
ENO1	TRINITY_DN76924_c1_g6	Alpha-enolase	7.645409467
TPI1	TRINITY_DN70056_c0_g3	Triosephosphate isomerase	3.876363706

### qRT-PCR analysis of key genes

**Fig 4:**
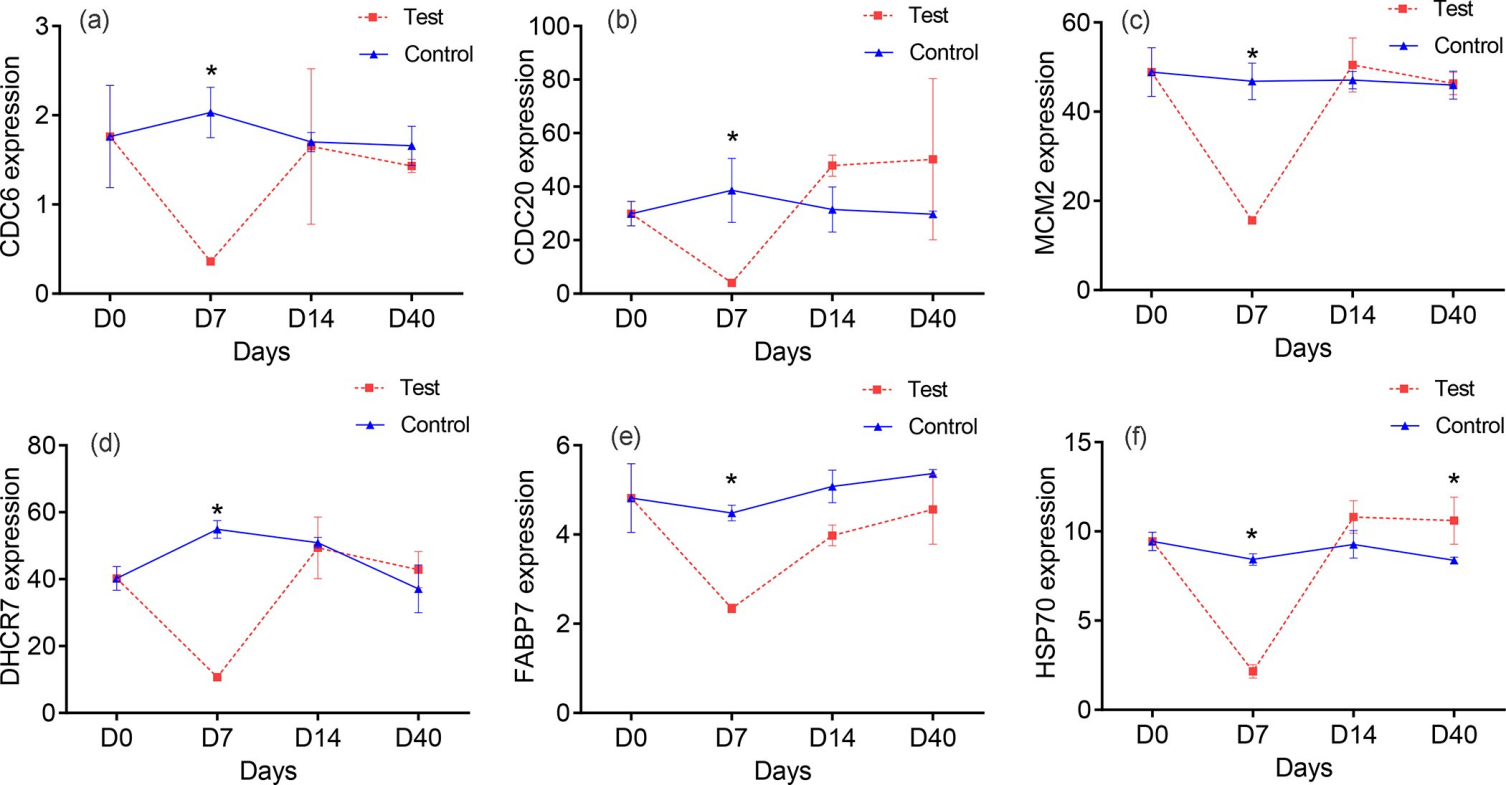
Analysis of the expression profiles of key genes using qRT-PCR. Asterisk indicates significant difference (*p* < 0.05), a~f represent the expression profiles of *CDC6*, *CDC20C*, *MCM2*, *DHCR7*, *FABP7*, and *HSP70*, respectively.

To investigate the expression profiles of key DEGs under the continuous feeding condition and fasting-refeeding condition, genes associated with cell cycle (*CDC6* and *CDC20*), DNA replication (*MCM2*), steroid biosynthesis (*DHCR7*), fat metabolism (*FABP7*), and stimulus response (*HSP70*) were analyzed using qRT-PCR. All of these genes (*CDC6*, *CDC20*, *MCM2*, *DHCR7*, *FABP7*, and *HSP70*) were significantly down-regulated after 7-day fasting. *CDC20* and *HSP70* were sharply increased and higher than the control group after 7-day refeeding. On day 33 of refeeding, *HSP7*0 remained significantly higher in the refeeding group than the control group. *CDC20*, however, was restored to normal level, and other genes were also restored and stable after refeeding. All genes had stable expressions under the continuous feeding condition (Fig 4).

### Confirmation of DEGs by qRT-PCR

**Fig 5:**
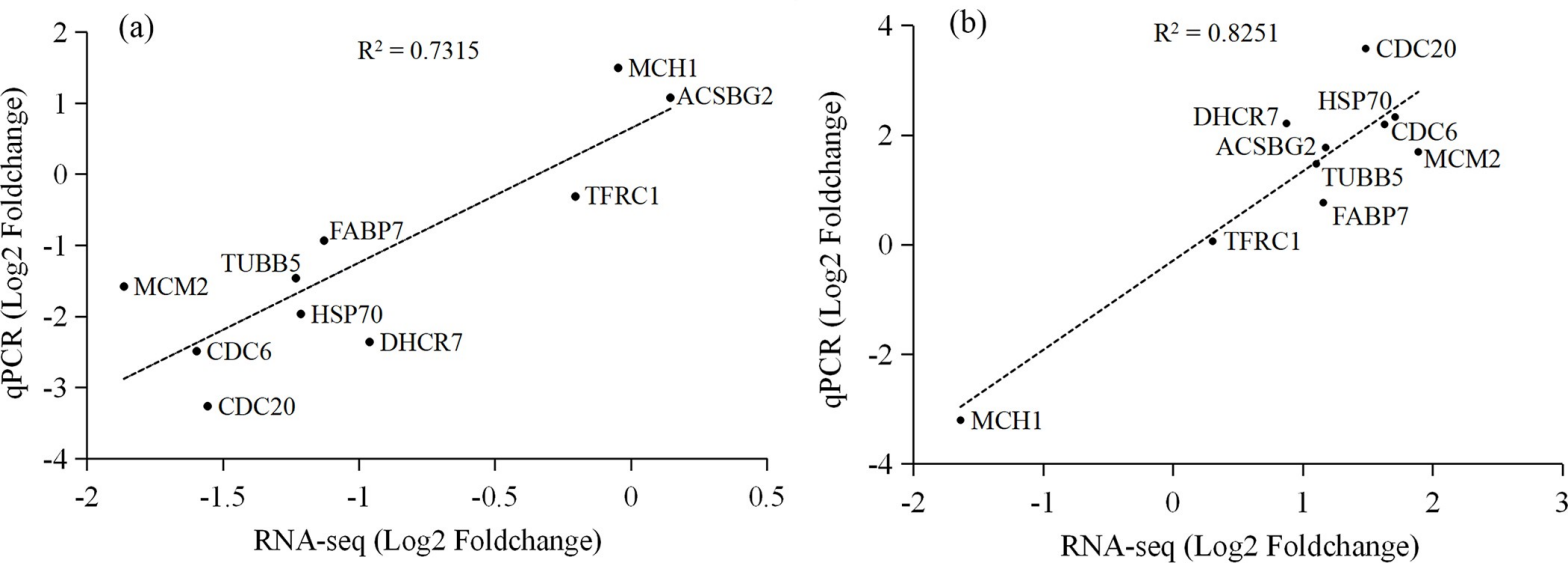
Correlation between results of RNA-seq and qRT-PCR. a and b represent correlations between control and fasting groups, and between control and refeeding groups, respectively. X-axis numbers represent the log_2_ (fold change) values from RNA-seq results. Y-axis numbers represent the log_2_ (fold change) values from qRT-PCR results.

To confirm the RNA-Seq results, 10 DEGs in different pathways were selected for qRT-PCR verification. Expression changes of these genes at fasting and refeeding were analyzed. The changes in gene expression obtained from qRT-PCR were consistent with the RNA-Seq under fasting and refeeding conditions, with R^2^ values of 0.7315 and 0.8251 (Fig 5), respectively. The results of the qRT-PCR analyses confirmed the reliability and accuracy of the data obtained by RNA-seq.

## Discussion

### 1. Changes in transcriptome profile of fish brain after 7-day fasting

The present study used RNA-Seq to investigate the impact of fasting and refeeding on transcriptome profiles of brain in *S*. *hollandi*. The global gene expression pattern of fish brain under continuous feeding condition, fasting condition, and refeeding condition were displayed. After 7-day fasting, 295 down-regulated and 169 up-regulated genes were identified in the brain.

The down-regulated DEGs were mainly enriched in the pathway of cell cycle, DNA replication, mitosis, steroid biosynthesis, immunity, gap junction, and sesquiterpenoid and triterpenoid backbone biosynthesis. Cyclins are important factors in the control of cell cycle, and can be activated by cyclin-dependent kinase enzymes [26]. Cyclin A2 (*CCNA2*), Cyclin-dependent kinase 1 (*CDK1*), and Cyclin-dependent kinase 2 (*CDK2*) were down-regulated during fasting. *CHEK2*, *E2F3*, and *ESPL1* play crucial roles in the control of cell cycle[27–29]. CDC20 is essential for the regulation of cell division; however, its most important function is to promote chromatid separation [30]. CDC6 is crucial to the maintenance of checkpoint mechanisms in the cell cycle and the loading of minichromosome maintenance (MCM) proteins onto DNA [31]. MCM, a DNA helicase composed of six subunits, is essential for genomic DNA replication. During fasting, *MCM2*, *MCM3*, *MCM4*, *MCM5-B*, and *MCM7-A* were all down-regulated. Mitosis is a part of the cell cycle wherein replicated sister chromatids are separated into two chromosomes. Several genes related to mitosis (*NCAPD2*, *NCAPG*, *PTTG1*, *SMC2*, and *SMC4*) were down-regulated. *NCAPD2*, *NCAPG*, *SMC2*, and *SMC4* were involved in chromosome condensation [32,33]. Securin, encoded by PTTG1, is a protein involved in the control of the metaphase-anaphase transition[34]. It has been widely reported that cell proliferation can be inhibited by starvation in mammal and fish [2,4]. The down-regulation of DEGs associated with steroid biosynthesis, immunity, and other functions may reduce energy expenditure and prolong the survival of *S*. *hollandi*.

Several genes associated with regulating glucose and lipid metabolism (*PDK4*, *ADIPOR1*, *ARRDC3*, *PMM1*, and *BBOX1*) were markedly up-regulated. *PDK4* decreases glucose utilization and increases fat metabolism through inhibiting pyruvate dehydrogenase. This plays an important role in regulating the shift in fuel economy to prolonged fasting and starvation [35]. *ADIPOR1* is essential in the regulation of normal glucose and fat homeostasis, and for maintaining normal body weight during the fasting period[36]. *ARRDC3* can decrease energy expenditure through decrease in the thermogenesis of adipose tissues [37] and *PMM1* has a phosphoglucomutase activity, which converts glucose-1-P into glucose-6-P[38]. BBOX1 is an enzyme involved in the biosynthesis of L-carnitine, a key molecule in fatty acid metabolism [39]. ZBTB16, a transcriptional repressor, plays a key role in various biological processes such as adipogenesis, regulation of lipid levels, and insulin sensitivity [40]. Maintaining metabolic homeostasis is essential for the survival of fish during fasting, and similar results have been reported in the rainbow trout, yellow croaker, and grass crap [3,14,41].

Three nutrition transport genes (*SLC43A2*, *SLC16A3*, and *SLC19A3*) were up-regulated. These trans-membrane proteins are substrate-specific and their increased expression may aid in the mobilization of nutrients. Several genes associated with ketone body metabolism (*SLC16A3* and *PDK4*) were up-regulated, and brain energy metabolism usually changes toward oxidation of ketone bodies during starvation [42]. Atrophy and autophagy can be induced by starvation, and protein synthesis can be inhibited. EEF2K can inhibit the eukaryotic elongation factor 2 (EEF2), an essential factor for protein synthesis. *FBXO32* is associated with muscle atrophy during the fasting period [43] and plays a similar function in brain. *GABARAPL1* and *TP53INP2* are essential genes for autophagosome maturation [44,45]. These DGEs can assist in alleviating starvation stress in fish. The change of transcriptome profiles associated metabolism and mobilization of stored energy were similar to the liver tissues [3].

### 2. Changes in transcriptome profiles of fish brain after refeeding

After refeeding, most DEGs down-regulated after fasting were restored to normal levels. Many of the genes associated with immunity and protein synthesis were up-regulated, and are known to play important roles in antigen processing and presentation, protein processing in endoplasmic reticulum and pathogenic *E*. *coli* infection. An improvement in disease resistance capability and immune status was observed in the refeeding fish. These fish were also seen to outperform the fish that were continuously fed.

Genes such as *ABCA1*, *ABCC5*, *IGFBP1*, *IRS2*, *JARID2*, *ACACB*, *CHKA*, *PDK2*, and *PPARA*, were down-regulated, implying these genes may not be key in fish recovery.

DEGs between control group and refeeding group were also analyzed. Many ribosomal proteins were over-expressed in the refeeding group. These proteins consisted of the ribonucleoprotein complexes and involved in the cellular process of translation. Stress-specific alterations in ribosomal proteins were reported in many species, the over-expressions of ribosomal proteins contributed to the starvation response. Similar result has been observed in the muscle of rainbow trout [46].

Several members of the heat shock proteins were over-expressed after refeeding. HSP is an important protein in response to the exposure to stressful conditions. Many members of HSP act as chaperones to assist new proteins, ensuring them correct folding and preventing protein damage by cell stress [47]. The over-expression of HSP can enhance translational efficiency and increase the ability of stimulus response.

Glycolysis and oxidative phosphorylation are the main metabolic pathways for releasing energy in organisms and provide energy for various physiological activities. The up-regulation of *LDHB*, *TPI1*, *GAPDH*, *PGAM1*, *LDHA*, *PGK1*, *ALDOA*, *GPI*, *MT-ND5*, and *MT-CO1* was beneficial for the production and restoration of energy.

In down-regulated DEGs, proopiomelanocortin (POMC) is an important precursor polypeptide, which can be enzymatically cleaved into many peptides, including melanotropins (α-MSH, β-MSH, and γ-MSH), adrenocorticotropin (ACTH), lipotropins, and endorphins [48]. These ligands closely associated with energy balance, glucose homeostasis and feeding behavior by negative regulation of energy metabolism [15,49]. The down-regulation of *POMC* is beneficial to diet intake.

### 3. Analyses of expression profiles of key DEGs using qRT-PCR

Expression profiles of six key DEGs were investigated using qRT-PCR. *CDC6*, *CDC20*, *MCM2*, *DHCR7*, and *FABP7* were down-regulated during fasting and restored after refeeding. DHCR7 plays a vital role in the cholesterol biosynthesis pathway, which is part of the steroid biosynthesis pathway [50]. Starvation inhibited steroid biosynthesis; thus, DHCR7 was down-regulated during fasting and restored after refeeding. FABP7 was down-regulated during fasting, and is most likely because the ketone body was the main energetic materials of brain during starvation. FABP3 was also down-regulated during fasting. *CDC20*, *CDC6*, and *MCM2* were all down-regulated because of the blockage of cell division by starvation. After refeeding, the expression of *CDC20* was higher than in the control group, despite there was no significant difference. HSP70 is important for protein folding, and protects cells from stress. Thus, the over-expression of *HSP70*, after refeeding, can protect organisms from undergoing stress.

## Conclusion

A transcriptome analysis was carried out to reveal the effects of fasting and refeeding on brains of juvenile *S*. *hollandi*. During fasting, the fish significantly reduced gene expression in the cell cycle, DNA replication, mitosis, steroid synthesis, and other physiological activities. After 7-day refeeding, the up-regulated DEGs were remarkably enriched in antigen processing and presentation, cell cycle, steroid biosynthesis, protein processing in endoplasmic reticulum, DNA replication, mitosis, pathogenic infection, and terpenoid backbone biosynthesis.

The study indicated that growth was inhibited during starvation since the fish mobilized the stored energy to prolong survival. After refeeding, the energy was stored again, and compensatory growth occurred, the body weight was recovered. In response to the starvation and feeding, a great deal of related genes and pathways were changed. This study provided the first transcriptome data on impacts of short-time starvation and refeeding to fish brains.

## Supporting information

S1 Fig Functional Gene Ontology classification (GO) of all unigenes. (TIF)

S2 Fig Cluster of Orthologous Groups of proteins (COG) of all unigenes. (TIF)

S1 Table Primers for quantitative real-time PCR. (DOCX)

S2 Table KEGG pathways for all unigenes. (DOCX)

S3 Table Up-regulated DEGs after fasting. (DOCX)

S4 Table Down-regulated DEGs after refeeding. (XLSX)

S5 Table Up-regulated DEGs between control group and fasting-refeeding group. (XLSX)

S6 Table Down-regulated DEGs between control group and fasting-refeeding group. (DOCX)
