# Supplementary figures and images for: Effect of fasting and subsequent refeeding on the transcriptional profiles of brain in juvenile Spinibarbus hollandi
Source: PLoS One. 2019 Mar 28;14(3):e0214589. doi: 10.1371/journal.pone.0214589 (PMC6438469; doi:10.1371/journal.pone.0214589)

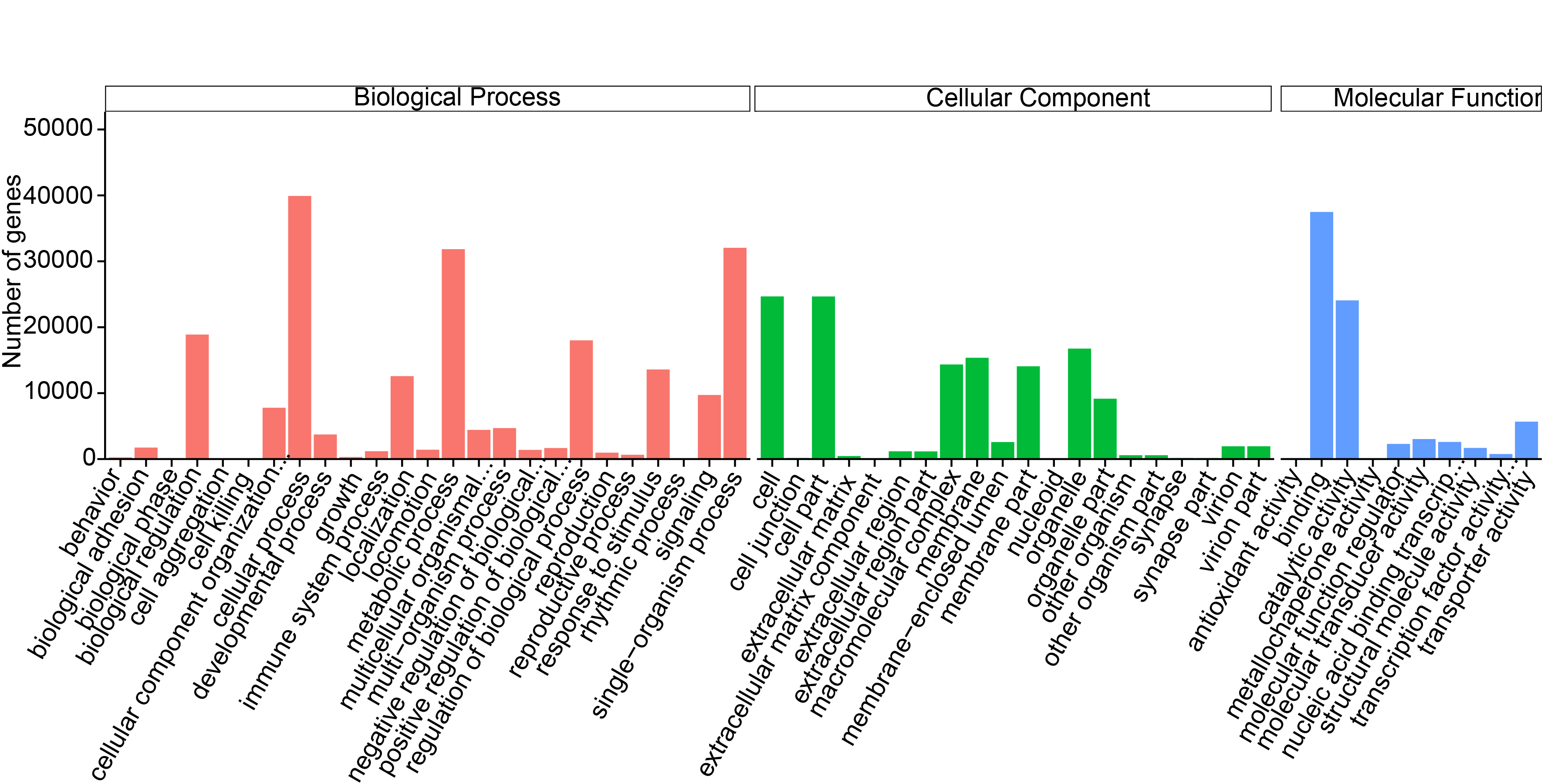

Supplement: S1 Fig — (TIF) [file pone.0214589.s001.tif]

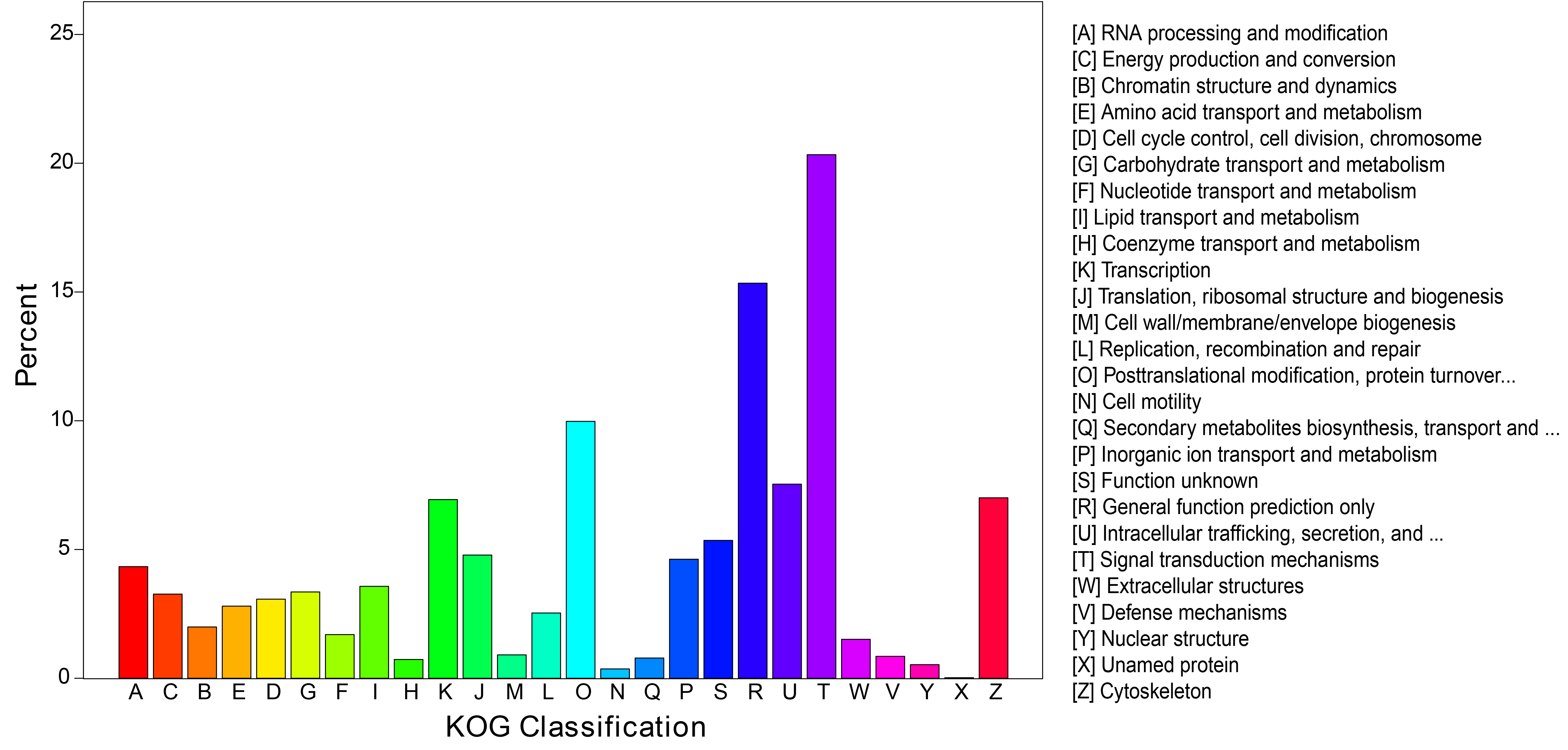

Supplement: S2 Fig — (TIF) [file pone.0214589.s002.tif]
