# Supplementary material for: Effect of fasting and subsequent refeeding on the transcriptional profiles of brain in juvenile Spinibarbus hollandi
Source: PLoS One. 2019 Mar 28;14(3):e0214589. doi: 10.1371/journal.pone.0214589 (PMC6438469; doi:10.1371/journal.pone.0214589)
Supplement: S1 Table — (DOCX) [file pone.0214589.s003.docx]

|  | Production length(nt) | primer (5'-3') | Primer length(nt) | Tm(℃) |
| --- | --- | --- | --- | --- |
| CDC20F | 116 | AGCACATCCAACTCCTCCAACACA | 24 | 60.8 |
| CDC20R |  | TTCTGGGTCTTGCCGTTCTTTCCT | 24 | 60.8 |
| CDC6F | 116 | TGAACTGCCGCTGCGTCCAA | 20 | 61 |
| CDC6R |  | TGCCGACCCAAACTGCTCAACT | 22 | 61 |
| DHCR7F | 129 | TTTGCTCTTGTGAATCCCGCCATC | 24 | 60.5 |
| DHCR7R |  | TTCCGCTCAACCAACCACCAGAA | 23 | 61 |
| FABP7F | 134 | ATGTTCCCTGTCACTCAAGCACCA | 24 | 60.8 |
| FABP7R |  | ACGCTCGGTCATCTGATTGGCTAG | 24 | 61.1 |
| HSP70F | 111 | GCGGTTTCAATACCCAGGGACAGA | 24 | 61.2 |
| HSP70R |  | GGCTTATGGTGCTGCGGTTCAAG | 23 | 61 |
| MCM2F | 123 | GAACGATCAGGACAGGACCAGCAT | 24 | 61 |
| MCM2R |  | ATAGGATTGGCAGCAGCGATGACA | 24 | 61 |
| TFRC1F | 100 | TGCCCAGCATTCCAGCCCAAA | 21 | 61 |
| TFRC1R |  | ATCCCATCACTCCAGCCATCAAGG | 24 | 60.8 |
| TUBB5F | 131 | GGTGCCAGGCTCCAGATCAACTAG | 24 | 61.3 |
| TUBB5R |  | GGATCGACCCAACAGGAACCTACC | 24 | 61 |
| MCH1F | 133 | CGTATGCATTCACGTCTGCG | 20 | 60 |
| MCH1R |  | AATGCTATATCCGCTCCCGC | 20 | 60.1 |
| ACSBG2F | 144 | TCCGGTTCAGCGAAATTGGT | 20 | 60.2 |
| ACSBG2R |  | CAGTGCAGTAGGTCGCATGA | 20 | 60.1 |
| ACTINF | 135 | TGACGGACAGGTCATCACCATTGG | 24 | 61.6 |
| ACTINR |  | GGTGGTCTCATGGATACCGCAAGA | 24 | 61.3 |

**S1 table. The primer pairs for qRT-PCR.**
