# Supplementary material for: Effect of fasting and subsequent refeeding on the transcriptional profiles of brain in juvenile Spinibarbus hollandi
Source: PLoS One. 2019 Mar 28;14(3):e0214589. doi: 10.1371/journal.pone.0214589 (PMC6438469; doi:10.1371/journal.pone.0214589)
Supplement: S2 Table — (DOCX) [file pone.0214589.s004.docx]

**S2 table. KEGG pathways for all unigenes.**

| KEGG signaling pathway | Pathway catalog | Pathway ID | Gene count |
| --- | --- | --- | --- |
| PI3K-Akt signaling pathway | Environmental Information Processing | ko04151 | 1350 |
| MAPK signaling pathway | Environmental Information Processing | ko04010 | 1182 |
| cAMP signaling pathway | Environmental Information Processing | ko04024 | 1141 |
| Calcium signaling pathway | Environmental Information Processing | ko04020 | 1041 |
| Rap1 signaling pathway | Environmental Information Processing | ko04015 | 1016 |
| Oxytocin signaling pathway | Organismal Systems | ko04921 | 878 |
| Adrenergic signaling in cardiomyocytes | Organismal Systems | ko04261 | 873 |
| Glutamatergic synapse | Organismal Systems | ko04724 | 762 |
| Axon guidance | Organismal Systems | ko04360 | 757 |
| Dopaminergic synapse | Organismal Systems | ko04728 | 749 |
| Purine metabolism | Metabolism | ko00230 | 633 |
| Carbon metabolism | Metabolism | ko01200 | 450 |
| Oxidative phosphorylation | Metabolism | ko00190 | 386 |
| Lysine degradation | Metabolism | ko00310 | 355 |
| Inositol phosphate metabolism | Metabolism | ko00562 | 321 |
| Protein processing in endoplasmic reticulum | Genetic Information Processing | ko04141 | 648 |
| RNA transport | Genetic Information Processing | ko03013 | 589 |
| Ubiquitin mediated proteolysis | Genetic Information Processing | ko04120 | 516 |
| Spliceosome | Genetic Information Processing | ko03040 | 489 |
| Ribosome | Genetic Information Processing | ko03010 | 400 |
| Focal adhesion | Cellular Processes | ko04510 | 1213 |
| Endocytosis | Cellular Processes | ko04144 | 1139 |
| Regulation of actin cytoskeleton | Cellular Processes | ko04810 | 1059 |
| Tight junction | Cellular Processes | ko04530 | 872 |
| Phagosome | Cellular Processes | ko04145 | 571 |
