# Supplementary material for: Effect of fasting and subsequent refeeding on the transcriptional profiles of brain in juvenile Spinibarbus hollandi
Source: PLoS One. 2019 Mar 28;14(3):e0214589. doi: 10.1371/journal.pone.0214589 (PMC6438469; doi:10.1371/journal.pone.0214589)
Supplement: S3 Table — (DOCX) [file pone.0214589.s005.docx]

**S3 table. Up-regulated DEGs after fasting.**

| Gene name | Gene ID | Gene description | log2FC |
| --- | --- | --- | --- |
| PDK4 | TRINITY_DN95752_c2_g2 | pyruvate dehydrogenase kinase isoenzyme 4 | 1.8265 |
| IRS2 | TRINITY_DN98427_c2_g1 | insulin receptor substrate 2 | 0.69336 |
| ADIPOR1 | TRINITY_DN92796_c0_g1 | Adiponectin receptor protein 1 | 0.7845 |
| ARL3 | TRINITY_DN89167_c2_g1 | ADP-ribosylation factor-like protein 3 | 0.60935 |
| ABTB1 | TRINITY_DN99943_c0_g1 | Ankyrin repeat and BTB/POZ domain-containing protein 1 | 3.1893 |
| KAL1 | TRINITY_DN111618_c6_g1 | Anosmin-1 | 1.1717 |
| ARRDC3 | TRINITY_DN107064_c1_g2 | Arrestin domain-containing protein 3 | 0.84685 |
| ARRDC3 | TRINITY_DN101818_c1_g2 | Arrestin domain-containing protein 3 | 0.89276 |
| KCNJ10 | TRINITY_DN102612_c4_g1 | ATP-sensitive inward rectifier potassium channel 10 | 0.68829 |
| KCNJ10 | TRINITY_DN98305_c4_g1 | ATP-sensitive inward rectifier potassium channel 10 | 0.60192 |
| PDE5A | TRINITY_DN95368_c1_g1 | cGMP-specific 3',5'-cyclic phosphodiesterase | 0.90502 |
| CBX7 | TRINITY_DN84769_c2_g1 | Chromobox protein homolog 7 | 0.62433 |
| STAG1 | TRINITY_DN102086_c1_g1 | Cohesin subunit SA-1 | 0.65182 |
| EEF2K | TRINITY_DN106107_c1_g1 | Eukaryotic elongation factor 2 kinase | 1.1637 |
| FBXO32 | TRINITY_DN93305_c1_g1 | F-box only protein 32 | 1.2457 |
| GABARAPL1 | TRINITY_DN75369_c2_g1 | Gamma-aminobutyric acid receptor-associated protein-like 1 | 0.86148 |
| BBOX1 | TRINITY_DN99777_c1_g1 | Gamma-butyrobetaine dioxygenase | 1.0218 |
| GLS | TRINITY_DN111620_c12_g1 | Glutaminase kidney isoform, mitochondrial | 0.70773 |
| SLC43A2 | TRINITY_DN107446_c2_g1 | Large neutral amino acids transporter small subunit 4 | 0.73614 |
| SLC16A3 | TRINITY_DN107827_c3_g1 | Monocarboxylate transporter 4 | 0.79206 |
| NCOA3 | TRINITY_DN109655_c2_g1 | Nuclear receptor coactivator 3 | 0.58425 |
| OAZ2 | TRINITY_DN89109_c0_g1 | Ornithine decarboxylase antizyme 2 | 0.71782 |
| PMM1 | TRINITY_DN101868_c1_g2 | Phosphomannomutase 1 | 1.0098 |
| PRDM5 | TRINITY_DN97197_c3_g1 | PR domain zinc finger protein 5 | 0.95255 |
| PRKCA | TRINITY_DN107660_c5_g2 | Protein kinase C alpha type | 0.57357 |
| TES | TRINITY_DN73167_c2_g1 | Testin | 4.0251 |
| TTC17 | TRINITY_DN108332_c1_g4 | Tetratricopeptide repeat protein 17 | 1.1058 |
| SLC19A3 | TRINITY_DN89387_c3_g3 | Thiamine transporter 2 | 0.94927 |
| TRIM16 | TRINITY_DN100388_c1_g1 | Tripartite motif-containing protein 16 | 0.93895 |
| TP53INP2 | TRINITY_DN98865_c0_g1 | Tumor protein p53-inducible nuclear protein 2 | 0.88086 |
| CACNG7 | TRINITY_DN86524_c1_g1 | Voltage-dependent calcium channel gamma-7 subunit | 0.71198 |
| ZBTB16 | TRINITY_DN106696_c2_g1 | Zinc finger and BTB domain-containing protein 16 | 0.76865 |
| ZNF778 | TRINITY_DN104036_c0_g1 | Zinc finger protein 778 | 1.577 |
