# Supplementary material for: Effect of fasting and subsequent refeeding on the transcriptional profiles of brain in juvenile Spinibarbus hollandi
Source: PLoS One. 2019 Mar 28;14(3):e0214589. doi: 10.1371/journal.pone.0214589 (PMC6438469; doi:10.1371/journal.pone.0214589)
Supplement: S6 Table — (DOCX) [file pone.0214589.s008.docx]

**S6 table. Down-regulated DEGs between control group and fasting-refeeding group.**

| Gene name | Gene ID | Gene description | log2FC |
| --- | --- | --- | --- |
| APP | TRINITY_DN16188_c0_g1 | Amyloid beta A4 protein | -3.61129 |
| ATP8B1 | TRINITY_DN90979_c4_g1 | Phospholipid-transporting ATPase IC | -2.88049 |
| CALM | TRINITY_DN83538_c0_g2 | Calmodulin | -4.25406 |
| CHMP4C | TRINITY_DN88324_c2_g3 | Charged multivesicular body protein 4c | -5.49517 |
| CPE | TRINITY_DN105318_c0_g3 | Carboxypeptidase E | -4.90509 |
| DES | TRINITY_DN97607_c2_g1 | Desmin | -3.12576 |
| FBRSL1 | TRINITY_DN98143_c2_g2 | Fibrosin-1-like protein | -0.79598 |
| FIBH | TRINITY_DN74789_c1_g1 | Fibroin heavy chain | -1.43493 |
| GAP43 | TRINITY_DN76306_c2_g6 | Neuromodulin | -4.32767 |
| GLUB5 | TRINITY_DN63920_c1_g1 | Glutelin type-B 5 | -7.94535 |
| GTF2IRD2 | TRINITY_DN109522_c2_g1 | General transcription factor II-I repeat domain-containing protein 2 | -4.55808 |
| JARID2 | TRINITY_DN104383_c0_g1 | Protein Jumonji | -1.45026 |
| JARID2B | TRINITY_DN91700_c1_g2 | Protein Jumonji | -1.17534 |
| KDM6B | TRINITY_DN100243_c1_g1 | Lysine-specific demethylase 6B | -1.0811 |
| KDM6B | TRINITY_DN108791_c4_g1 | Lysine-specific demethylase 6B | -0.9027 |
| KIF21A | TRINITY_DN92964_c8_g1 | Kinesin-like protein KIF21A | -4.05961 |
| MRC2 | TRINITY_DN74880_c2_g3 | C-type mannose receptor 2 | -3.50584 |
| MT-CO3 | TRINITY_DN100593_c1_g1 | Cytochrome c oxidase subunit 3 | -10.1754 |
| MT-ND3 | TRINITY_DN86326_c3_g1 | NADH-ubiquinone oxidoreductase chain 3 | -2.75158 |
| MT-ND6 | TRINITY_DN73743_c5_g1 | NADH-ubiquinone oxidoreductase chain 6 | -8.07228 |
| NCOA3 | TRINITY_DN73831_c0_g1 | Nuclear receptor coactivator 3 | -0.85155 |
| NNAT | TRINITY_DN69959_c0_g1 | Neuronatin | -5.17144 |
| NPAS4 | TRINITY_DN107376_c1_g1 | Neuronal PAS domain-containing protein 4 | -1.31793 |
| NPAS4 | TRINITY_DN107376_c1_g2 | Neuronal PAS domain-containing protein 4 | -1.04451 |
| PDE9A | TRINITY_DN91497_c0_g1 | High affinity cGMP-specific 3',5'-cyclic phosphodiesterase 9A | -1.5057 |
| POMCA | TRINITY_DN80017_c0_g1 | Pro-opiomelanocortin-1 | -3.30213 |
| PPM1H | TRINITY_DN91146_c3_g1 | Protein phosphatase 1H | -2.88651 |
| PTMS | TRINITY_DN97540_c2_g2 | Parathymosin | -6.11827 |
| RPH3A | TRINITY_DN71643_c0_g2 | Rabphilin-3A | -1.96729 |
| RTN1 | TRINITY_DN61472_c0_g1 | Reticulon-1 | -4.31657 |
| STMN2 | TRINITY_DN92575_c2_g9 | Stathmin-2 | -5.00059 |
| TUBB2A | TRINITY_DN82523_c2_g6 | Tubulin beta-2A chain | -4.43129 |
| UCHL1 | TRINITY_DN84304_c2_g5 | Ubiquitin carboxyl-terminal hydrolase isozyme L1 | -4.03735 |
| WDR35 | TRINITY_DN110544_c3_g3 | WD repeat-containing protein 35 | -4.43336 |
| ZNF10 | TRINITY_DN98111_c2_g2 | Zinc finger protein 10 | -0.72047 |
| ZNF184 | TRINITY_DN102865_c1_g1 | Zinc finger protein 184 | -0.77022 |
